# Genomic variants in mouse model induced by azoxymethane and dextran sodium sulfate improperly mimic human colorectal cancer

**DOI:** 10.1038/s41598-017-00057-3

**Published:** 2017-02-07

**Authors:** Qingfei Pan, Xiaomin Lou, Ju Zhang, Yinghui Zhu, Fuqiang Li, Qiang Shan, Xianwei Chen, Yingying Xie, Siyuan Su, Hanfu Wei, Liang Lin, Lin Wu, Siqi Liu

**Affiliations:** 1 CAS Key Laboratory of Genome Sciences and Information, China Gastrointestinal Cancer Research Center, Beijing Institute of Genomics, Chinese Academy of Sciences, Beijing, China; 2 Sino-Danish Center for Education and Research, University of Chinese Academy of Sciences, Beijing, China; 3 BGI-Shenzhen, Shenzhen, China; 4 Beijing Protein Innovation, Beijing, China

## Abstract

Mouse model induced by azoxymethane (AOM) and dextran sodium sulfate (DSS) is generally accepted as an ideal object to study on the carcinogenesis mechanisms of human colorectal cancer (CRC). The genomic responses to the AOM/DSS treatment in mouse that possibly lead to elucidation of CRC pathological mechanism are still poorly understood. For the first time, we investigated the cancer genome landscape of AOM/DSS mouse model by exome sequencing, to testify its molecular faithfulness to human CRC. Of 14 neoplastic samples, 7575 somatic variants were identified, which resulted in 2507 mutant genes and exhibited a large diversity in both colorectal aberrant crypt foci (ACF) and tumors even those tissues that were gained from the similar morphology or same treatment period. Cross-species comparison of the somatic variants demonstrated the totally different patterns of variable sites, mutant genes and perturbed pathways between mouse and human CRC. We therefore come to a conclusion that the tumorigenesis at genomic level in AOM/DSS model may not be properly comparable with that in human CRC, and the molecular mechanism elicited from this animal model should be carefully evaluated.

## Introduction

Human cancer studies are limited by many ethical and practical considerations^1^. Animal model, by contrast, is believed to be an ideal alternative for its convenience in controlling experimental conditions, monitoring pathological development, achieving enough materials and avoiding ethical risks. An appropriate model is of great significance in cancer mechanism research, diagnosis and therapeutic evaluation. First reported in 1996^2^, AOM/DSS mouse model was proven as an outstanding model in faithfully mimicking of the pathological changes of “normal-ACF-adenoma-carcinoma” development^3^ as observed in human CRC^4^, and hence was widely used in studies of colorectal carcinogenesis and chemopreventive intervention.

As regards an appropriate cancer model, in addition to similar pathological phenotypes it is highly expected that the model also carries comparable molecular features and follows same carcinogenesis progress with its cognate human cancer type^5^. Several investigations focusing on the molecular features of AOM/DSS model were implemented, which reported some common features shared by human and mouse CRC, such as mutation or deregulation of *KRAS* ^6^ and *CTNNB1* ^7,8^. Besides, high-throughput technologies were also adopted in exploration of molecular features in this model at both transcriptional and translational level. Using DNA microarray technique, Suzuki *et al.* discovered hundreds of differentially expressed genes between 5 and 10 weeks of AOM/DSS treatment^9^. With a similar strategy, Li *et al.* claimed that abundances of both mRNA and miRNA were impacted by AOM/DSS^10^. At protein level, Yasui *et al.* identified 21 differential proteins between tumorous and their adjacent tissues in the animal model using two-dimensional gel electrophoresis and mass spectrometry^11^. However, in spite of these works, molecular mechanism of AOM/DSS mouse model is still very limited. Firstly, all the high-throughput data was acquired from the tumor/normal mixed tissues or the pooled samples from individuals. Generally, the strategy of which the tumor/normal pairs are individually analyzed enables production of more convincing data. Secondly, the studies based on traditional techniques like PCR and IHC only offered partially molecular responses to AOM/DSS, which those observations often conflicted lab to lab. For instance, the distinct mutation frequencies of *Kras* and different mutation sites of β-catenin were documented from several labs^12,13^. A global and systematical estimation towards the genomic responses to AOM/DSS in mouse could establish an overall feature that benefits a reasonable comparison of pathological genomics between mouse and human CRC.

Cancer is believed a disease with genomic defect^14^. Cancer genomics based on next-generation sequencing (NGS) provides an informative resource for characterization of cancer-related genes and identification of novel genetic alterations, and contributes a critical step towards understanding of cancer initiation, progression and metastasis^15^. Several studies have suggested that sequencing tumor genomes of animal models is also an effective approach for discovering the gene mutations in mouse that are mimic human malignancies. In the example of *Trp53*-mutated breast cancer mouse model, some key features associated with the cognate human tumors were found, such as the homozygous in-frame deletion of *Lrp1b* even though with huge diversity of somatic rearrangements^16^. By sequencing tumor-normal paired samples from a mouse model mimicking the acute promyelocytic leukemia (APL), Wartman *et al.* revealed a somatic *Jak1* ^*V657F*^ mutation in the same residue of *JAK1* as previously found in human APL^17,18^. However, overall evaluation towards the variants at genomic level has been not reported yet in AOM/DSS mouse model.

In this study, we employed whole exome sequencing to explore the genome landscapes of neoplastic samples with different morphologies or from different phases of tumorigenesis in AOM/DSS mouse model. For the first time, we revealed the cancer genome landscape of both ACF and tumors in this model, and systematically compared of the variable sites, mutant genes and perturbed pathways of CRC between mouse and human. On the basis of the overall evaluation for the faithfulness of this model to human CRC, we concluded that the patterns of genomic variants in AOM/DSS were somehow different from that chronically developed in human CRC.

## Results

### Evaluation of AOM/DSS mouse model

**Figure 1:**
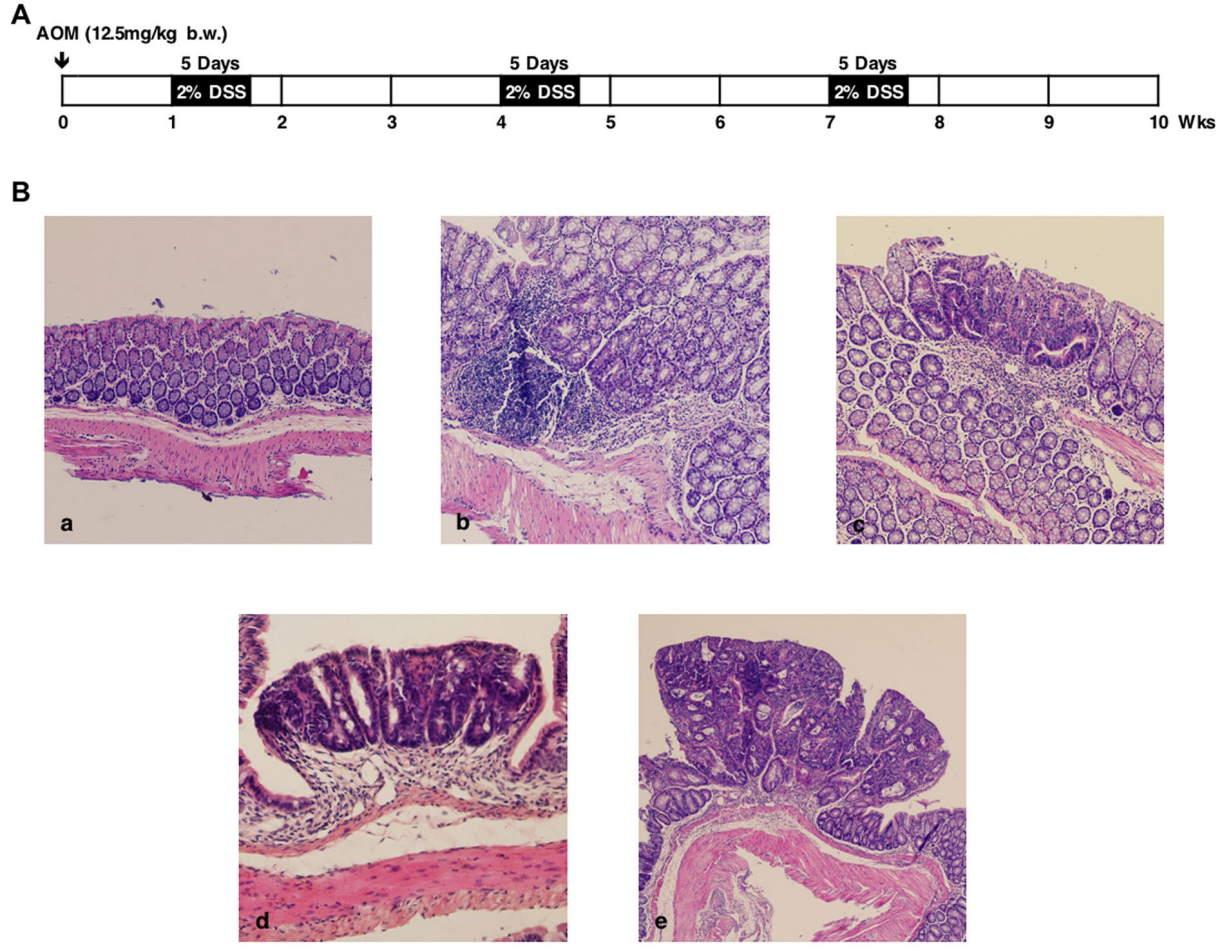
Establishment and histopathological evaluation of AOM/DSS mouse model. (**A**) Schedule of model establishment. (**B**) Histopathology of colonic dysplasia in different phases of model establishment: a) normal colorectal mucosa, x10; b) colorectal mucosa, 4 weeks of AOM/DSS treatment, x10; c) ACF, 7 weeks of AOM/DSS treatment, x10; d) adenoma, 7 weeks of AOM/DSS treatment, x20; e) adenocarcinoma, 10 weeks of AOM/DSS treatment, x4.

The AOM/DSS mouse model were assessed in five aspects during its establishment. 1) Tumor formation. Under light microscope tubercles were rarely observed in control mice, whereas a number of tubercles were found in all treated mice, mainly in distal part of colorectums (Supplementary Fig. S1A). Tubercles in the mice with two DSS cycles were quite different from those with three DSS cycles in both number and size, 5.8 ± 2.0 tubercles per mouse with 1.9 ± 0.9 mm in size for the former, and 12.2 ± 2.6 per mouse with 3.2 ± 1.6 mm for the later. 2) Bloody diarrhea. This symptom was absent in control mice, but began to appear in treated mice at the first DSS cycle at a low incidence of 8%. With increased DSS cycles, it became more and more serious, reaching 36% and 79% at the second and third DSS cycle, respectively. 3) Anorectal prolapse. This symptom was only found in the third DSS cycle with 43% incidence (Supplementary Fig. S1B). 4) Loss of body weight. In contrast to control mice whose body weights remained continuously increasing during the establishment, all treated mice underwent a typical cycle of body weight change in each DSS feeding cycle, in which their body weights reduced in response to DSS feeding and recovered in about 10 days (Supplementary Fig. S1C). And 5) Histopathological properties. The histopathological properties related to CRC development were examined by HE staining (Fig. 1B). Compared with the normal mucosa (Fig. 1B-a), serious lymphocytes infiltration appeared on the mucosa layer in the mice with one DSS cycle (Fig. 1B-b), which was a typical inflammation sign. The presence of ACF (Fig. 1B-c) and adenoma (Fig. 1B-d) in colorectal mucosa was widely observed in mice with two DSS cycles, while the corresponding area exhibited the cellular atypia, such as larger and darker nucleus, enhanced polarity of nucleus and increased ratio of nucleus to cytoplasm. The significant adenocarcinoma (Fig. 1B-e) was perceived in the mice with three DSS cycles, with more and more cellular and architectural atypia. A multistep process, following “normal-ACF-adenoma-carcinoma” sequence, was clearly observed. The above evaluations firmly indicated that AOM/DSS mouse model was well established.

### Somatic variants in AOM/DSS mouse model

**Figure 2:**
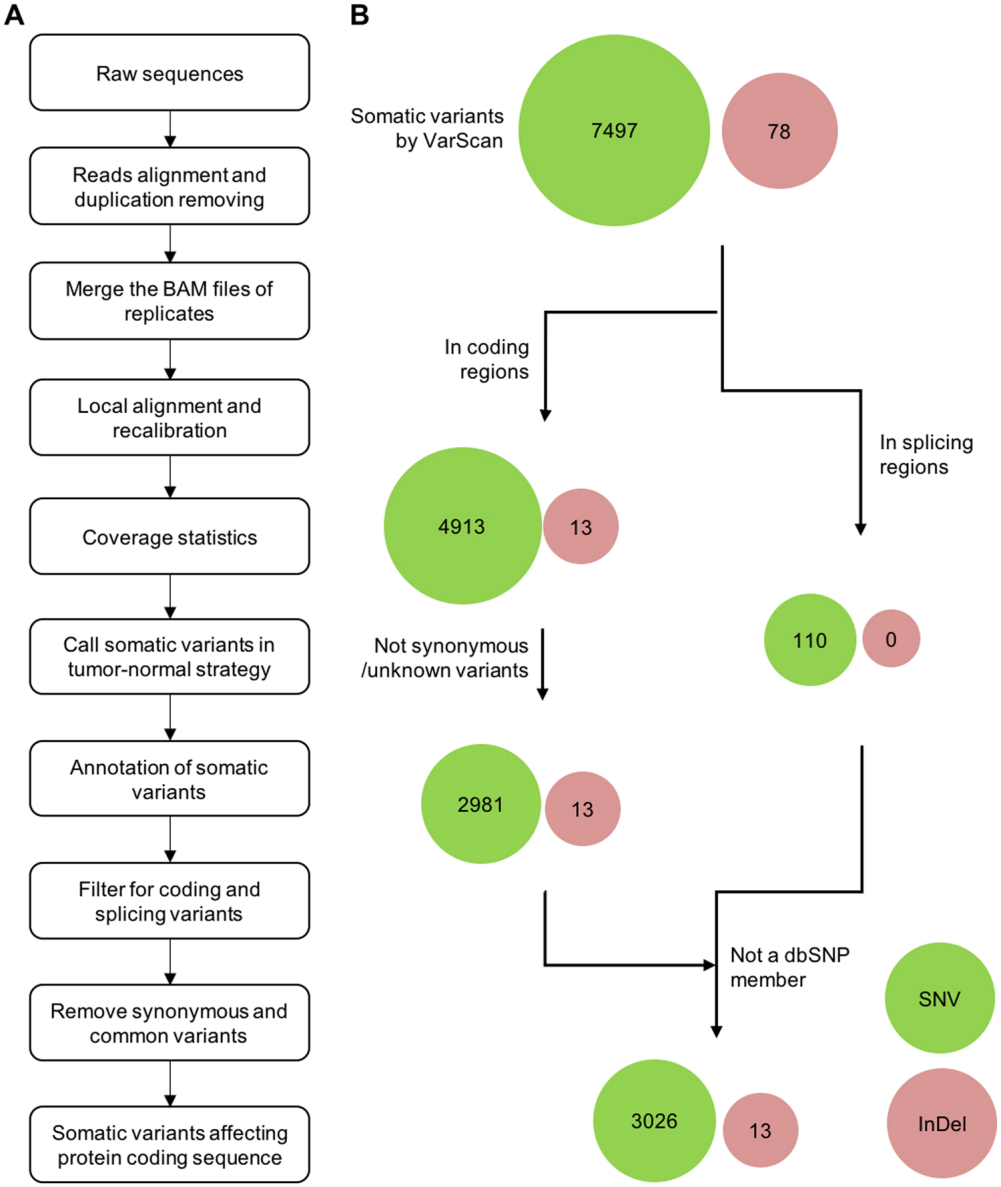
Workflow and filtering strategy for identifying somatic variants affecting protein coding sequence. (**A**) Following the filtration of low-quality reads, all of the qualified reads were aligned to the mouse reference genome with BWA. Duplicated reads were removed and the results of two technical replicates were merged by SAMTools. After the local alignment and recalibration by GATK, the somatic variants of neoplastic samples were called by VarScan2 and further filtered by self-built Perl scripts. (**B**) A cumulative filtration strategy was introduced to identify the somatic variants affecting protein coding sequence. Somatic variants located in coding or splicing regions were achieved according to the annotation results. Those variants annotated as synonymous and unknown by ANNOVAR, as well as those present in dbSNP, were filtered out. With this filtration strategy, 7497 SNVs and 78 InDels were defined as somatic variants in AOM/DSS mouse model, of which 3026 SNVs and 13 InDels were predicted as protein-changing variants. Circles representing SNVs and InDels are colored lawngreen and indianred, respectively.

A workflow (Fig. 2A) was conducted to identify the somatic variants in AOM/DSS mouse model. In all the mouse samples sequenced in this study, only one set of sequencing data (7-5-A) was disqualified in quality control, thus it was removed in next data analysis. The sequencing data was statistically evaluated towards the rest 24 samples, which resulted in the sequencing depth of 209.0 ± 29.6 in SureSelect target regions and 194.1 ± 26.6 folds in Consensus Coding Sequence (CCDS), and the sequence coverage of over 97% at 8x and over 94% at 20x against both regions (Supplementary Table S2). Somatic variants in each neoplastic sample were identified with a tumor-normal paired strategy by removing the variants in their paired-normal samples, and the variants affecting protein coding sequence were further filtrated step by step (Fig. 2B). The data analysis revealed that a total of 7575 somatic variants were identified in 14 neoplastic samples from AOM/DSS mice (Supplementary Table S3), including 1863 silent, 2860 missense, 121 nonsense substitutions, 110 splice-site mutations and 2543 mutations in UTR, intronic and intergenic regions, in addition to 78 small insertions or deletions (InDels). Using Sequenom platform, 250 somatic variants were randomly selected and 237 (95%) of them were confirmed, implying that the somatic variants generated from exome sequencing and data analysis were highly accepted.

**Figure 3:**
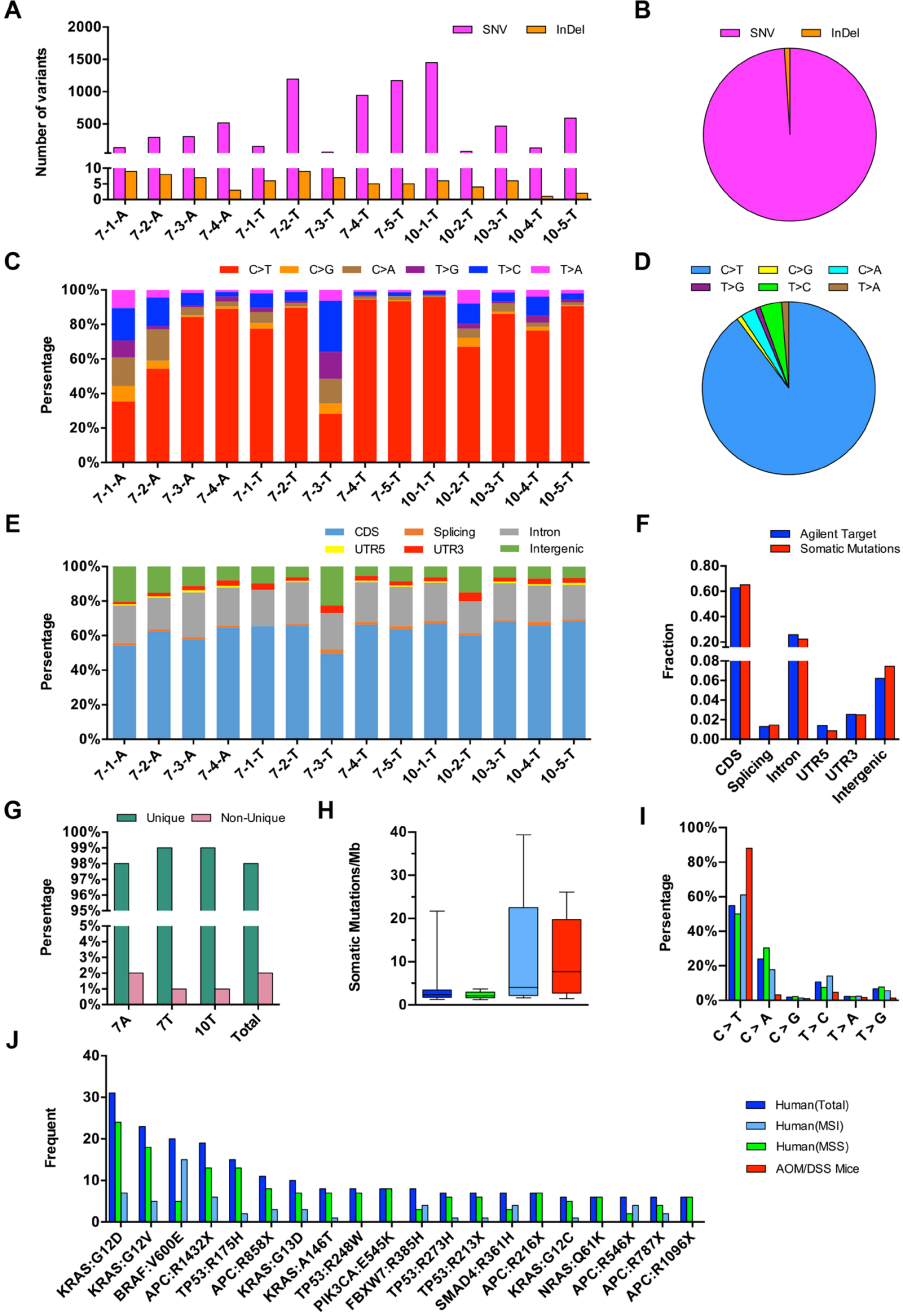
Landscape of somatic variable sites in AOM/DSS mouse model and its comparisons with Human CRC. (**A**) Numbers of somatic SNVs and InDels in each of 14 neoplastic samples. (**B**) The ratio of SNVs to InDels in the total somatic variable sites is showed with pie plot. (**C**) Relative proportion of the six mutational spectra in each neoplastic sample. (**D**) The proportion of six mutational spectra in the total somatic variable sites is showed with pie plot. (**E**) Relative proportion of somatic variants in each of six functional regions as indicated in the legend box in each of samples. (**F**) Proportions of total somatic mutations (blue) and capture-targeting sites (red) in six functional regions. (**G**) Percentage values of sample-unique somatic variable sites and non-sample-unique somatic variable sites in group 7A, 7T, 10T and total of 14 neoplastic samples. (**H**) Box plot shows the range of mutation rates in three groups of human CRC and AOM/DSS mice. (**I**) Percentage values of six mutational spectra in three groups of human CRC and AOM/DSS mice. (**J**) Frequents of the top 20 most frequently mutated sites in human CRC reported by TCGA in three groups of human CRC and AOM/DSS mice. In Fig. 3**H**, (**I** and **J**), three groups of human CRC, Total, MSS and MSI, are indicated with blue, green and sky blue, respectively. The group of AOM/DSS mice is indicated with red.

The specifically genomic characteristics of these somatic variants were summarized as follows. Firstly, huge diversity of somatic variants was found among individuals in both number and content. Among the 14 neoplastic samples, the number of somatic variants ranged from 71 (1.4 per Mb) to 1457 (28.6 per Mb), with 393 (7.7 per Mb) and 541 (10.6 per Mb) as the median and mean respectively (Fig. 3A). In addition, these somatic variants were poorly shared by these 14 samples. Even though the samples within the same groups which had similar morphologies, over 98% of their somatic variants were individual-unique (Fig. 3G). Secondly, the structure variants were rarely observed in these samples which contained only 73 (1%) variants as small insertions or deletions, whereas the others (99%) were single-nucleotide substitutions (Fig. 3B). Based on a Circular Binary Segmentation (CBS) method, the copy number variants in AOM/DSS mouse model was investigated, and no consistent segment with significant copy number changes was found (Supplementary Fig. S3). Thirdly, with a percentage of 90%, the mutation spectrum of substitutions was dominated by C:G > T:A, which was consistent with the features of AOM-based induction (Fig. 3C and D). And fourthly, the somatic variants were unlikely to be enriched in certain functional regions, as the proportions of somatic mutations were similar in all the different functional regions in the whole capture region (Fig. 3E and F).

As regards an animal model, we questioned how AOM/DSS mouse model was faithfully mimic human CRC. To answer this question, we collected the exome sequencing data of human CRC by The Cancer Genome Atlas (TCGA) and divided the data into three groups, Total that contained all the cases (224), MSS (microsatellite stability) with 159 cases, and MSI (microsatellite instability) with 64 cases. And the cross-species analysis on the genomic variants of mouse and human CRC was implemented. At the site level, three features of genomic variants were comprehensively compared. 1) The average mutation rate of AOM/DSS mouse model (10.6 per Mb) was larger than that of MSS cases (6.7 per Mb), but smaller than that of MSI cases (16.9 per Mb). The range of mutation rates was a critical parameter to assess genomic stability. As shown in Fig. 3H, its value of MSS cases was much smaller than that of MSI cases and AOM/DSS mice, implying that the genomic instability of AOM/DSS was basically comparable to MSI. 2) Because of the strong effect of AOM, the proportion of C:G > T:A transitions in mouse (90%) was much higher than that of human groups (67%, Fig. 3I). And 3) We selected the top 20 most frequently mutated sites among TCGA samples, all of which were located within the driver genes of CRC including *APC*, *TP53*, *KRAS*, *NRAS*, *BRAF*, *PIK3CA*, *SMAD4* and *FBXW7*, and mapped them to the mouse exome sequencing data. Surprisingly, none of them was detectable in AOM/DSS mice (Fig. 3J). Collecting all the comparisons mentioned above, the evidence supported the observation that these somatic variable sites in response to AOM/DSS in mice were distinct from that in human CRC.

### Mutant genes in AOM/DSS mouse model

**Figure 4:**
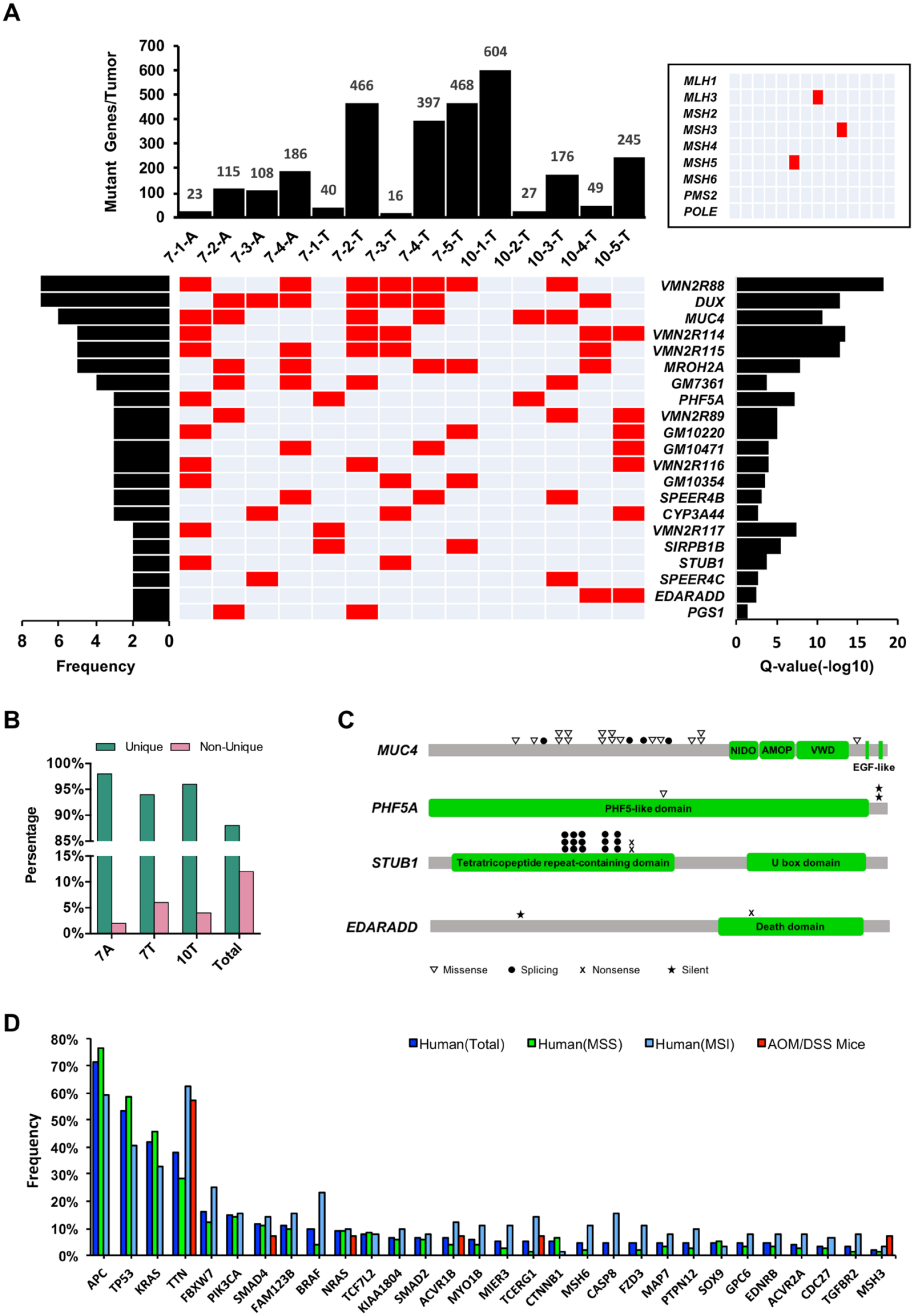
Landscape of somatic mutant genes in AOM/DSS mouse model and its comparisons with Human CRC. (**A**) Number of somatic mutant genes in each of 14 neoplastic samples are indicated with bar plot. The recurrent SMGs (P-value < 0.01 and Q-value < 0.1) identified in AOM/DSS mouse model are listed vertically by frequent and Q-value. A heat map, in which a red square indicated mutation of specific SMG in corresponding sample, show the distribution of such SMGs in 14 neoplastic samples. By the left, the frequent of individuals with mutations of each gene is shown with bar plot, while by the right is the Q-value of each SMG. Another heat map in the upper right corner shows the mutational profiling of nine MMR genes in AOM/DSS mouse model. (**B**) Percentage values of sample-unique somatic mutant genes and non-sample-unique somatic mutant genes in group 7A, 7T, 10T and total of 14 neoplastic samples. (**C**) Mapping of somatic mutations in four cancer-related SMGs, MUC4, PHF5A, STUB1 and EDARADD. (**D**) Frequencies of the 30 SMGs of human CRC reported by TCGA in three groups of human CRC and AOM/DSS mice. Three groups of human CRC, Total, MSS and MSI, are indicated with blue, green and sky blue, respectively, while the group of AOM/DSS mice is indicated with red.

Totally, 2507 mutant genes (Supplementary Table S4) were defined from 2986 somatic mutations affecting protein coding sequences (1.19 mutations per gene), with the median in 145 and mean in 209 per sample, respectively. The somatic mutant genes per sample were quite diverse, however, ranging from 16 to 604 (Fig. 4A). And these mutant genes were poorly overlapped among all samples (<11%, Fig. 4C) and different samples within each of three groups (<6%, Fig. 4B). The DNA mismatch-repair (MMR) genes, such as *MLH1*, *MLH3*, *MSH2*, *MSH3*, *MSH4*, *MSH5*, *MSH6*, *PMS2* and *POLE*, were reported widely mutated in hypermutated cases of human CRC. These genes were carefully examined in this mouse model, and a few of mutated MMR genes with low frequencies were found in these samples. The huge genome instability caused by AOM/DSS treatments appeared independent from mutations of MMR genes.

The significantly mutated genes (SMGs) were evaluated by DrGap, which is an efficient tool to define the SMGs in many cancer types without species limitations^19^. With this tool, 25 out of 2507 somatic mutant genes were defined as SMGs with *P* < 0.01 and a false discovery rate (FDR) *Q*-value < 0.1 (Supplementary Table S4), and 21 of them were recurrent among the 14 neoplastic samples (Fig. 4A). Further exploration on the functions of these recurrent SMGs showed that most of them (17/21) were not reported yet as cancer-related genes. As regards the other four SMGs, *Phf5a* ^20^, *Stub1* ^21^, *Edaradd* ^22^ and *Muc4* ^23–26^, all of them were reported to be associated with carcinogenesis. Then we checked the distribution of their somatic mutations, and found most of the somatic mutations on *Muc4*, *Phf5a* and *Edaradd* out of functional domains (Fig. 4C), suggesting those mutations not fundamentally important to the gene functions. These somatic mutations of the four SMGs were further evaluated by SIFT, resulting in most of them as tolerated but not deleterious. Based on these analysis, the performance of such SMG-based strategies in explaining the mechanisms of carcinogenesis was very poor in this chemical-induced mouse model.

An evitable issue for mouse model studies is the evaluation of faithfulness of AOM/DSS mouse model in mimicking the pattern of somatic mutant genes in human CRC. We firstly compared the profiling of somatic mutant genes between mouse and human (Supplementary Fig. S4A and B). A poor overlap statistics was observed between mouse and human CRC, which could be explained by the huge difference of case number between these two species. To eliminate this bias, only shared genes were taken into subsequent correlation analysis. Heat map of these shared genes (Supplementary Fig. S4C and D) indicated the different patterns of mutation frequency between mouse model and human CRC. Pearson correlations (Supplementary Fig. S5) were introduced to quantify the difference among the four groups and the result showed that correlation efficiencies (*R* ^2^) between human groups were ranged from 0.46 to 0.84, whereas 0.07 to 0.08 between mouse and human groups. Huge differences of mutant gene frequencies between mouse and three human groups were indeed perceived. And secondly, we sought the overlap rate of the human CRC SMGs between mouse and human groups. As shown in Fig. 4D, 30 SMGs in human CRC^27^ were no overlap with the SMGs in AOM/DSS mouse model. If the overlap comparison is extended to the human CRC SMGs and all the somatic mutant genes in AOM/DSS mice, only 6 human SMGs were overlapped with the mouse model at low frequency, approximately 7%. Clearly, the key mutated genes related with human CRC were totally different from the somatic mutant genes in AOM/DSS mice. Besides, some highly mutated genes in human CRC, such as *APC*, *TP53*, *KRAS* and *PIK3CA*, were rarely identified in this model. Taken all the mutant gene comparisons, we came to a conclusion that the somatic mutant genes induced by acute treatment of AOM/DSS in mouse might be different from that caused by chronically pathological changes in human colorectal mucosa.

### Frequently perturbed pathways in AOM/DSS mouse model

**Figure 5:**
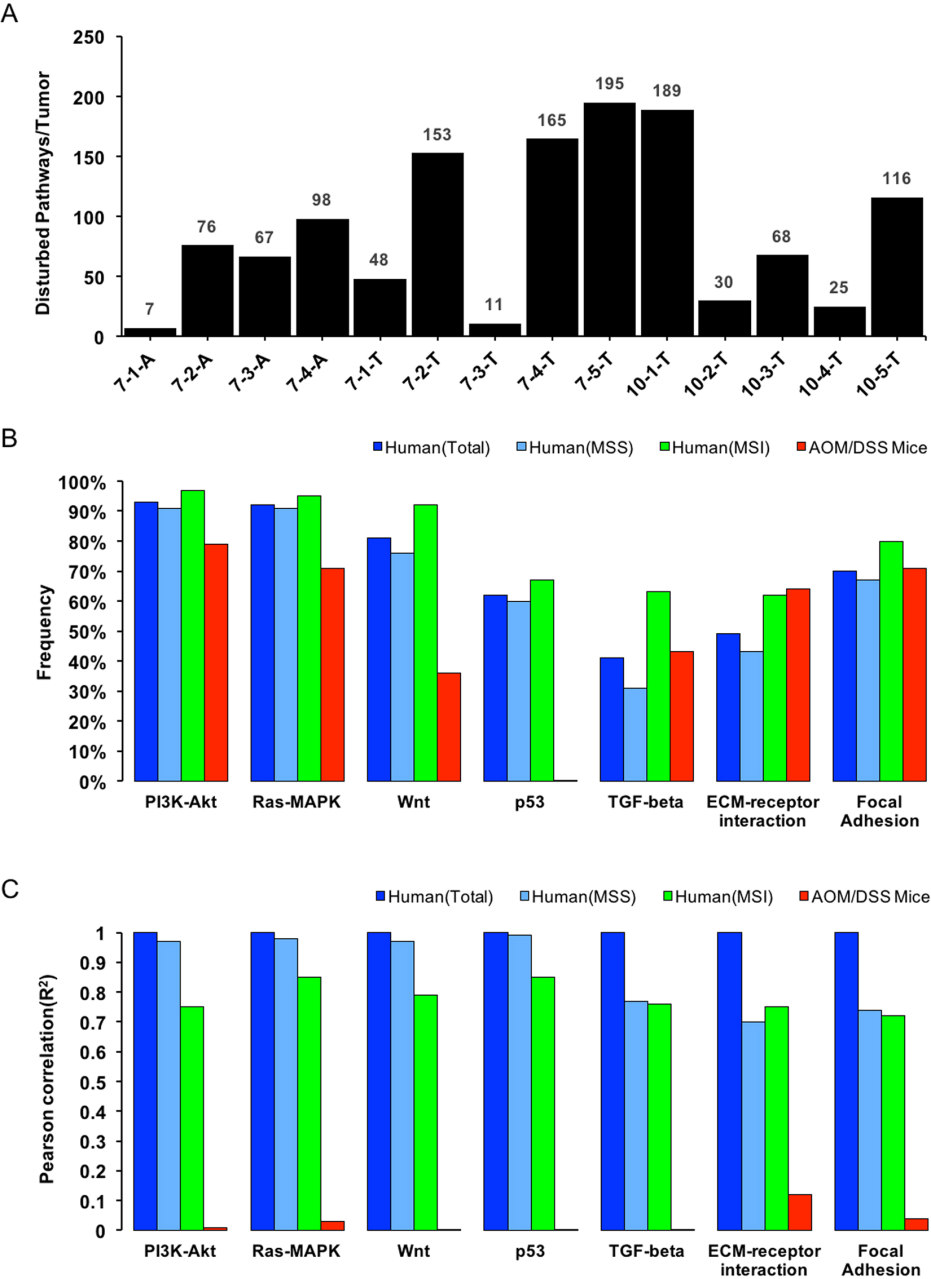
Landscape of disturbed pathways in AOM/DSS mouse model and its comparisons with Human CRC. (**A**) Number of disturbed pathways in each of 14 neoplastic samples is indicated with bar plot. Comparisons of five key pathways in human CRC and two most enriched pathways in AOM/DSS mouse model among three groups of human CRC and AOM/DSS mice in their frequencies (**B**) and patterns of damaging genes involved (**C**). In Fig. 5C, shown are bar graphs of Pearson correlation (R^2^) for the seven pathways. The groups of total Human CRC cases is shown as the reference.

Frequently perturbed pathway is a critical concept to study the mechanisms of tumorigenesis. To assess the pathways potentially perturbed in AOM/DSS mouse mode, 1523 somatic mutant genes with “damaging” mutations predicted by SIFT, were defined as candidates and acquired for subsequent analysis. Based on these damaging genes, the frequencies of disturbed pathways in these samples were estimated followed by an enrichment analysis (Methods). The analysis resulted in an extensive disturbance of pathways ignited by AOM/DSS: 264 out of 290 (91%) KEGG pathways (Supplementary Table S5) were disturbed and 63 perturbed pathways were found in at least half of all samples. Besides, the number of perturbed pathways among different individuals were also of huge diversity, with a range from 7 to 195 per sample (Fig. 5A). If a strict criterion for pathway enrichment with *P* < 0.01 and FDR < 0.1 was taken, total of 21 pathways (Supplementary Table S5) were significantly enriched. Ranking the enriched pathways upon FDR, two pathways at the top were functionally oriented to ECM-receptor interaction and focal adhesion, implying that the genes involved in cell-cell adhesion might play important roles in carcinogenesis of this model.

We further pursued whether the profiling of disturbed pathways in AOM/DSS mouse model was similar to that in human CRC. To avoid the bias in the definitions of KEGG pathways in mouse and human, we converted 1523 damaging genes in the mouse and gained a new dataset containing 1359 human homologous genes. With the same strategy as those used in gene level, we first globally evaluate the similarity of disturbed pathway frequencies between AOM/DSS mouse model and each of three human groups. As shown in Supplementary Fig. S6, the Pearson correlations (*R* ^2^) between mouse and human (0.40, 0.36 and 0.50) were much smaller than that between different groups of human CRC (0.99, 0.93 and 0.86).

There were five core pathways related with human CRC found by TCGA^28^. As mentioned above, two adhesion-related pathways were highly enriched in AOM/DSS. We then focused on comparison of these pathways between mouse and human, for their perturbed frequencies and patterns of damaging genes. Of the seven pathways for comparison, only two pathways, Wnt and p53 signaling, exhibited the obviously different perturbed frequencies between mouse and human (Fig. 5B), while the other five pathways between the two species were basically comparable in perturbed frequency. This parameter seemed not sensitive enough to draw any conclusion from the comparison. Further comparison was conducted to the pattern of damaging genes involved in the seven pathways between AOM/DSS mice and human CRC. The damaging gene frequency to certain pathways was calculated in human CRC and AOM/DSS mice, and the correlations of damaging gene frequencies in each of the seven key pathways between the two species were evaluated and indexed with Pearson correlations (*R* ^2^). As shown in Fig. 5C, there were the satisfactory correlations of damaging gene frequencies among the three human CRC groups, whereas was a huge difference of such correlation between AOM/DSS mice and human CRC, regardless of Total, MSS and MSI groups. For example, the perturbed frequency of PI3K-Akt signaling pathway was over 90% in human CRC, and was about 80% in AOM/DSS mice. Look at the top three somatic mutant genes of this pathway in human CRC, there were *TP53* with 44.6% in Total, 49.1% in MSS, and 32.8% in MSI, *KRAS* with 41.5% in Total, 45.3% in MSS and 32.8% in MSI and *PIK3CA* with 12.5% in Total, 11.9% in MSS and 14.1% in MSI, however, none of them was detected in AOM/DSS mice. The correlation analysis towards this pathway between the two species in Fig. 5C showed a poor correlation efficiency of 0.01. Therefore, the correlative assessment of damaging gene frequency in pathways demonstrated that the pathways alternated responding to AOM/DSS treatment in mice were not properly comparable with the pathways affected by chronically pathological developments in human.

## Discussion

As a cornerstone of modern biomedical research, especially in the field of cancer study, mouse is a dominant resource in animal modeling. In this study, we proposed that comparison analysis of genomics between normal and neoplastic tissues in AOM/DSS mouse model could offer a novel view to explore the tumorigenesis mechanism. At genomic view, a set of mice from the same strain is believed to share quite similar genetic background so that these mice are expected to have comparable genomic responses to a same stimulus. Moreover, during construction of AOM/DSS model, the mouse gender, age and drug inducement could be well controlled. The biological heterogeneity among mice is reasoned to be significantly narrow down. Therefore, we did not introduce a large scale animal experiment but placed five mice in each experimental group to evaluate the genomic responses to AOM/DSS. The exome sequencing results described above gave us a shock. Although the genomic alternations in the mice without drug treatment or the colorectal tissues adjacent to tumor remained little, the genomic responses to AOM/DSS in the ACF and CRC tissues were found in a huge diversity even though these tissues shared with the similar morphology or treated with the same drug and time. Further cross-species comparison revealed a distinct pattern of the somatic variants between mouse and human CRC. How do we explain the phenomenon?

The genomic structures of mouse, such as telomere crisis^29^, transformation requirements^30^ and centromere distribution^31^, are very distinct from that in human. These differences are likely to make the specifically genomic features in the colorectal tumors of mouse. For instance, Wartman *et al.* generated a mouse model of APL and surprisingly found significant genetic variation from the tumor sample compared with that of the mouse reference genome^32^. Of over thirty thousand potentially somatic variants, only 3 somatic and nonsynonymous mutations were finally confirmed to be associated with human APL. Varela and his colleagues established genetically engineered mouse models of breast cancer and employed paired-end sequencing to identify the somatic structural variants involved in the carcinogenesis in the model^16^. A huge diversity of somatic variants was perceived in that model due to heterogeneity of somatic rearrangement and no recurrent fusion gene was found. To evaluate the faithfulness of such models in mimicking human inflammatory diseases, Seok *et al.* made three murine injury models, burn, trauma and endotoxemia, and profiled the gene expression in such models^33^. Correlations of the inflammatory-dependent genes by Pearson correlations (*R* ^2^) revealed low correlation rates at *R* ^2^ = 0.00–0.13 between mouse and human. Another intriguing phenomenon was that even *APC*, *P53* and *KRAS* with highly somatic mutations in human CRC have been widely observed, these genes did not show any mutation in this study. When the rat CRC was generated by AOM/DSS, *Apc* and *Kras* mutations were identified at the frequency of 8%^34^ and 30–60%^35–37^, respectively, whereas no mutation of *p53* was reported^35^. When the mouse CRC was constructed by AOM/DSS in other labs, there was no mutation found in *Apc* and *p53* ^38^, while very low mutation frequency (0–10%) of *Kras* was detected^8,39^. Our results were in a good agreement with these observations, of which the gene mutations provoked by AOM/DSS are species dependent. These studies upon genomics analysis towards to disease mouse model has demonstrated the genomic stability in mouse quite different from that in human. Our study provided another evidence to support the hypothesis that a mouse model may follow a unique pathogenesis, at least at genomic level.

Another concern to evaluate the differently genomic responses between mouse and human CRC is the drug treatment with AOM and DSS. First of all, the tumorigenesis mechanisms in AOM/DSS mouse was reported not to fully recapitulate all the cancer subtypes of human CRC. According to the general protocol of AOM/DSS mouse modeling, the model only properly mimics to inflammatory bowel disease (IBD)-related CRC^40^. As a matter of fact, the IBD-related CRC just accounts for 1–2% of all human CRC^41^. As the somatic mutations generated from the human CRC genomics analysis were not only focused on the IBD-related CRC, the mutation patterns responsive to AOM/DSS was reasonable not very comparable with the generally genomic mutations in human CRC. Secondarily, AOM is a carcinogen that causes base mispairings by inducing *O* ^*6*^-methylguanine adducts in guanine^40^. Considering the ubiquitous distribution of guanine, the distribution of genomic variants caused by AOM is theoretically random, while our sequencing data (Fig. 3F) was in a good agreement with the theoretical anticipation. There is no standard protocol globally accepted for construction of AOM/DSS mouse model. To generate an appropriate model, we have referenced several important publications in the field, and paid an attention to the technique details, specifically in dose usage and cycling time of drug inducement. Upon our knowledge, the protocol described in this paper could represent a general approach in AOM/DSS model construction, and could guarantee the colorectal tumor development within ten weeks after drug treatment. As illustrated in Figs 3 and 4, however, somatic variable sites or mutant genes in the mouse colorectal tissues presented a huge diversity which was independent from morphology and/or treatment period. Our exome sequencing data, therefore, raised a question whether the general approach proper to explore the CRC initiation factors at genomic level. Under such strong drug stress, the driver gene mutations might be merged into the huge signals of the passenger mutations. In further study, re-evaluation to the genomic responses of mouse colorectal tissues under different doses and treatment periods is helpful to explore the driver genes to mouse CRC induced by AOM/DSS.

In summary, for the first time the rationality of AOM/DSS mouse model is challenged by the exome sequencing evidence obtained from the colorectal tumor tissues. Although at morphological level, the CRC development in the mice after AOM/DSS inducement is basically mimic to human one, the somatic mutations appeared a completely different pattern between mouse and human CRC. Moreover, the diversity of somatic mutations among the pathological tissues of mouse implied that the treatment of AOM/DSS triggered a wide instability in mouse genome. The experimental evidence thus leads to two clues in further study, whether genomic analysis is a valuable means to evaluate a disease animal model and the gene expression at large scale in AOM/DSS model is necessary to be done in near future.

## Methods

### Establishment of AOM/DSS mouse model

To well control the animal source, we set the strict criteria for mouse selection, in which all the mice for AOM/DSS model construction should come from C57BL/6N inbred strain within the same brood, male, age with 7 weeks and body weight ranged 18–20 g (Vital River, Inc., Beijing). Total of 45 mice were selected, 10 as controls without any treatments and 35 as experimentals treated with AOM/DSS. We followed a well-accepted protocol^42^ to establish the AOM/DSS mouse model using two drugs: azoxymethane (AOM, CAT NO: A5486 from Sigma-Aldrich Co.) and dextran sodium sulfate (DSS, M.W. 36000 to 50000, CAT NO: 160110 from MP Biomedicals). As shown in Fig. 1A, after one week’s adaptive phase in animal house, mice in treated group received a single intraperitoneal injection of AOM with a dose of 12.5 mg/kg body weight. One week later, the mice were given two or three cycles of DSS treatment. Each cycle lasts for 3 weeks, with 2% DSS-dissolved water for 5 days and tap water for 16 days. The animals in the control group were untreated. During the model establishment, the physical signs, like body weight, bloody diarrhea and anorectal prolapse, were tracked and recorded. Meanwhile, two treated mice were sacrificed in week 1, 4, 7 and 10 respectively and conducted to histopathological examination to evaluate the quality of mouse model we built. Our study was approved by the ethics committee of Beijing Institute of Genomics, Chinese Academy of Sciences, Beijing, China. All experiments were performed in accordance with relevant guidelines.

### Sample preparation

Mice were sacrificed in week 7 and 10 for sample preparation. A methylene blue based method without fixation was developed and evaluated (Supplementary Fig. S2). After sacrifice, the large intestine of each mouse was cut open longitudinally, and washed with phosphate buffered saline (PBS). The distal part of colorectum (~2 cm from the anus) was collected for next operations: 1) the collected distal colorectum were fully spread on clean filter paper, with the lining of intestine facing up. The tubercles were counted and their sizes were measured; 2) the colorectum were stained by fresh methylene blue (2% in PBS, w/v) at 37 °C for 25 minutes with shaking at 70 r/min; 3) after staining, the samples were destained by fresh PBS at 37 °C with shaking at the same speed. During the process of destaining, the destaining solutions should be changed frequently, and the progress of detaining should be detected every 5 minutes by microscopic inspection. The destaining should be stopped when the crypts could be identified clearly by microscope; and 4) the tumors and ACFs were isolated by micro-dissecting instruments under the microscopic view. In total, six groups of samples were collected from 3 kinds of mice: 1) for mice in control group, normal mucosa were collected as group 0C; 2) for mice from treated group with 2 DSS cycles, ACFs (termed as group 7A), small tumors (7T), and their adjacent normal mucosa (7N) were collected; and 3) for mice from treated group with 3 DSS cycles, tumor (10T) and adjacent normal mucosa (10N) were collected. All samples were stored in liquid nitrogen at −80 °C before DNA extraction.

### Whole genome amplification, exome capturing and sequencing

25 samples from five groups were selected for exome sequencing (Supplementary Table S1). Genomic DNA was extracted using the QIAamp DNA Micro Kit (CAT NO: 56304) and quantified through Qubit Fluorimeter (Invitrogen, USA). For each sample, two aliquots of 10 ng of gDNA were used as technical replicates. The resulting 50 samples were processed to whole genome amplification with REPLI-g Mini Kit (CAT NO: 150025). All the amplified products passed the quality control: 1) concentration: >37.5 ng/μl; 2) total amount: >3 μg; and 3) length of main electrophoresis band: >10 Kb. Then paired-end libraries of the 60 amplified samples were prepared (PE-91, Illumina, San Diego, CA, USA). Exome sequences were captured with SureSelect^XT^ Mouse All Exon Kit (G7550E-001, Agilent, CA, USA) following the standard protocols. The products of exome capture should pass these criteria: 1) the length of fragments: 300 ± 30 bp; and 2) total amount: >600 ng. After exome capturing, the index-tagged samples were pooled and sequenced on Illumina HiSeq 2000 with 90 or 100 bp length.

### Read mapping and detection of somatic variants

Burrows-Wheeler Alignment (v-0.7.4) was employed to align reads to the reference genome (mm9/NCBIM37) with default parameters. Duplicated reads were removed and the BAM files of technical replicates were merged by SAMTools (v-0.1.19). After local realignment and recalibration by the Genome Analysis Toolkit (v-1.6-13-g91f02df), the coverage on both SureSelect target regions and the CCDS were calculated. Sample with <90% coverage in these two regions at 20x were filtered out. Somatic variants were call by VarScan2 (v-2.3.6) in tumor-normal pairs and filtered with self-built Perl scripts. Criteria for somatic variants included: 1) read depth is at least 8 in tumor and normal; 2) minimal mean of base quality is 15; 3) minimal allele frequencies are 0.12 for SNVs and 0.2 for InDels; 4) *p*-value to call a heterozygote is <0.05; 5) *p*-value to call a somatic site is <0.01; 5) supported by both plus and minus strands, and the ratio of supporting reads in one strand should be no more than 95%.

### Validation of somatic variants

Somatic variants detected in AOM/DSS mouse model were validated by Sequenom Mass ARRAY genotyping assays. Somatic variants were randomly selected from 14 diseased samples followed by primer design using Assay Design Suite v2.0, a free online tool (https://www.mysequenom.com/Tools). Native DNAs were used as templates in most samples, and for those with insufficient native DNA, DNA products from whole genome amplification of both replicates were used. Signals (Peaks) were counted as positive only if they were detected in both replicates. Only those variants detected in tumor samples and undetected in adjacent normal samples were regarded to be successfully validated. Variants failed in validation were filtered out before next analysis.

### Somatic variants annotation

ANNOVAR (Revision:514)^43^ were introduced in annotation of somatic variants based on refGene database (updated in Nov. 2014)^44,45^. Self-built Perl scripts were used to annotate the existence of somatic variants in dbSNP (v128) and filter for those somatic variants affecting protein coding sequence. We further evaluated the effect of somatic variants on protein functions by SIFT annotation with PROVEAN^46^. The Kyoto Encyclopedia of Genes and Genomes (KEGG, http://www.kegg.jp, updated in Apr. 27^th^, 2015)^47,48^ was used to define the altered pathways in each sample. LiftOver^49^ was used to transfer the coordinates of somatic variants from mm9 to mm10.

### Establishment of significantly mutated genes

Significantly mutated genes in AOM/DSS mouse model were predicted by a computational tool named DrGap (Driver Genes and Pathways)^19^ with default settings. The gene mutation table of mm9, which is necessary in this analysis but not supplied by the author, was calculated and summarized by Perl scripts based on the information supplied by the refGene database which was used in the annotation of somatic variants. This table was available in Supplemental Table S6.

### Enrichment analysis of KEGG pathways

A hypergeometric distribution analysis (Fisher’s Exact Test) followed by a correction for multiple testing (Bejamini-Hochberg FDR correction) was used to determine the pathways overrepresented in AOM/DSS mouse model. In order to remove passengers as much as possible, only “damaging” genes (with nonsynonymous mutations predicted as “damaging” by SIFT, mutations leading to stop codon gains and losses, mutations in splicing regions, and somatic insertions and deletions located in both coding and splicing regions) were used in this analysis. Pathways with less than 10 members or more than 500 members were excluded. All of the 24062 genes collected in refGene database were used as a background gene list. Perl code-based scripts were used in this analysis for data format conversion, summarization and reporting.

### Cross-species analysis of tumors between AOM/DSS mouse model and human CRC

Exome sequencing data of 224 cases by TCGA, including 169 MSS cases and 64 MSI cases, were collected for the cross-species analysis as the representative of human CRC dataset. Combining the dataset of AOM/DSS mouse model, four groups were derived for cross-species analysis: 1) Human (Total) which including all of the 224 human CRC cases; 2) Human (MSS) which including 169 MSS cases; 3) Human (MSI) which including 64 MSI cases and 4) AOM/DSS Mice which including the 14 cases we sequenced. The cross-species analysis was performed on three levels: variable sites, mutated genes and altered pathways. To avoid the bias in the definitions of KEGG pathway between mouse and human, the damaging genes of AOM/DSS mouse model were converted to human homologs with NCBI homology database. Self-built Perl scripts were used to calculate the frequencies of variable sites, mutated genes and altered pathways in each of the four groups for further comparison, analyze the overlap among different groups and prepare formatted data for visualization. R script were used in the calculation of Pearson Correlation (*R* ^2^) and data visualization.

### Data access

The sequence data from this study have been submitted to the NCBI Sequence Read Archive (SRA; http://www.ncbi.nlm.nih.gov/sra/). Accession numbers are listed in Supplementary Table S7.

## Electronic supplementary material

Supplementary PDF File

Supplementary Table S3

Supplementary Table S4

Supplementary Table S5

Supplementary Table S6
