# Erratum: Genomic variants in mouse model induced by azoxymethane and dextran sodium sulfate improperly mimic human colorectal cancer

**DOI:** 10.1038/s41598-017-00076-0

**Published:** 2017-06-01

**Authors:** Qingfei Pan, Xiaomin Lou, Ju Zhang, Yinghui Zhu, Fuqiang Li, Qiang Shan, Xianwei Chen, Yingying Xie, Siyuan Su, Hanfu Wei, Liang Lin, Lin Wu, Siqi Liu

**Affiliations:** 1 CAS Key Laboratory of Genome Sciences and Information, China Gastrointestinal Cancer Research Center, Beijing Institute of Genomics, Chinese Academy of Sciences, Beijing, China; 2 Sino-Danish Center for Education and Research, University of Chinese Academy of Sciences, Beijing, China; 3 BGI-Shenzhen, Shenzhen, China; 4 Beijing Protein Innovation, Beijing, China

## Abstract

A correction to this article has been published and is linked from the HTML version of this paper. The error has been fixed in the paper.

*Scientific Reports* **7**:25; doi:10.1038/s41598-017-00057-3; Article published online 07 February 2017

The original version of this Article stated an incorrect publication date. In addition, the Acknowledgments section of this Article was incomplete:

This project is financially supported by the National Natural Science Foundation of China (NO. 91131009). We thank Beijing Protein Innovation for their help in animal model establishment, BGI-Shenzhen for exome capturing and sequencing, and Beijing Institute of Genomics for the validation of somatic mutations.

Now reads:

This project is partially supported by the National Natural Science Foundation of China (NO. 91131009 and NO. 81372601), and the Fundamental & Advanced Research Project of Chongqing, China (NO. cstc2013jcyjC00001). We thank Beijing Protein Innovation for their help in animal model establishment, BGI-Shenzhen for exome capturing and sequencing, and Beijing Institute of Genomics for the validation of somatic mutations.

These errors have now been corrected in the HTML and PDF versions of the Article.

