# Supplementary material for: Genomic variants in mouse model induced by azoxymethane and dextran sodium sulfate improperly mimic human colorectal cancer
Source: Sci Rep. 2017 Feb 7;7:25. doi: 10.1038/s41598-017-00057-3 (PMC5453956; doi:10.1038/s41598-017-00057-3)
Supplement: Supplementary file 1 — Supplementary PDF File [file 41598_2017_57_MOESM1_ESM.doc]

**Genomic variants in mouse model induced by azoxymethane and dextran sodium sulfate improperly mimic human colorectal cancer**

Qingfei Pan,^1,2^ Xiaomin Lou,^1^ Ju Zhang,^1^ Yinghui Zhu,^1^ Fuqiang Li,^3^ Qiang Shan,^1^ Xianwei Chen,^1^ Yingying Xie,^1^ Siyuan Su,^1^ Hanfu Wei,^4^ Liang Lin,^3^ Lin Wu^1,2^*, and Siqi Liu^1,2,3^*


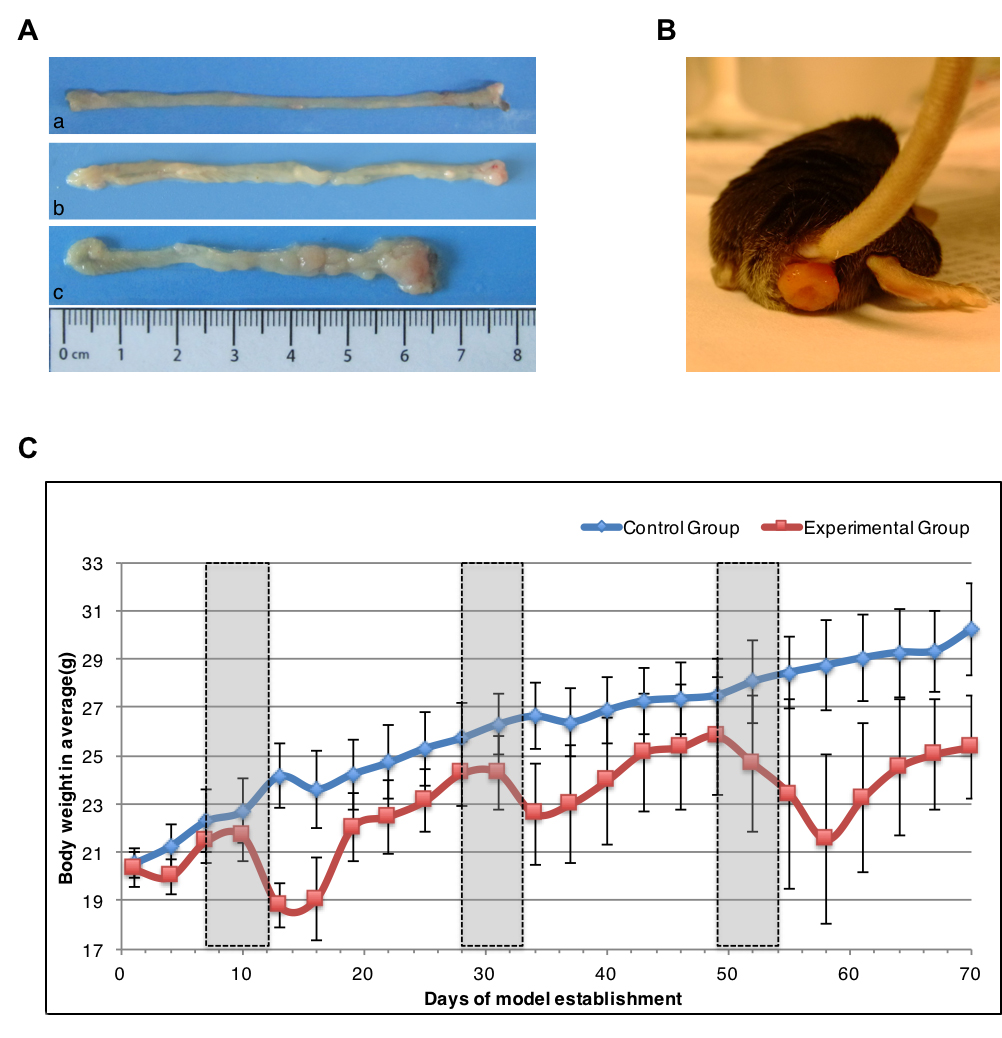


**Supplemental Figure S1. General observations of AOM/DSS mouse model.** (A) Macroscopic view of the colorectum of mice with different AOM/DSS treatments: a) no treatment; b) AOM injection and 2 DSS feeding cycles; and c) AOM injection and 3 DSS feeding cycles. (B) Anorectal prolapse. (C) The dynamic change of body weight with AOM/DSS treatments. Periods of DSS feedings are marked with grey boxes.


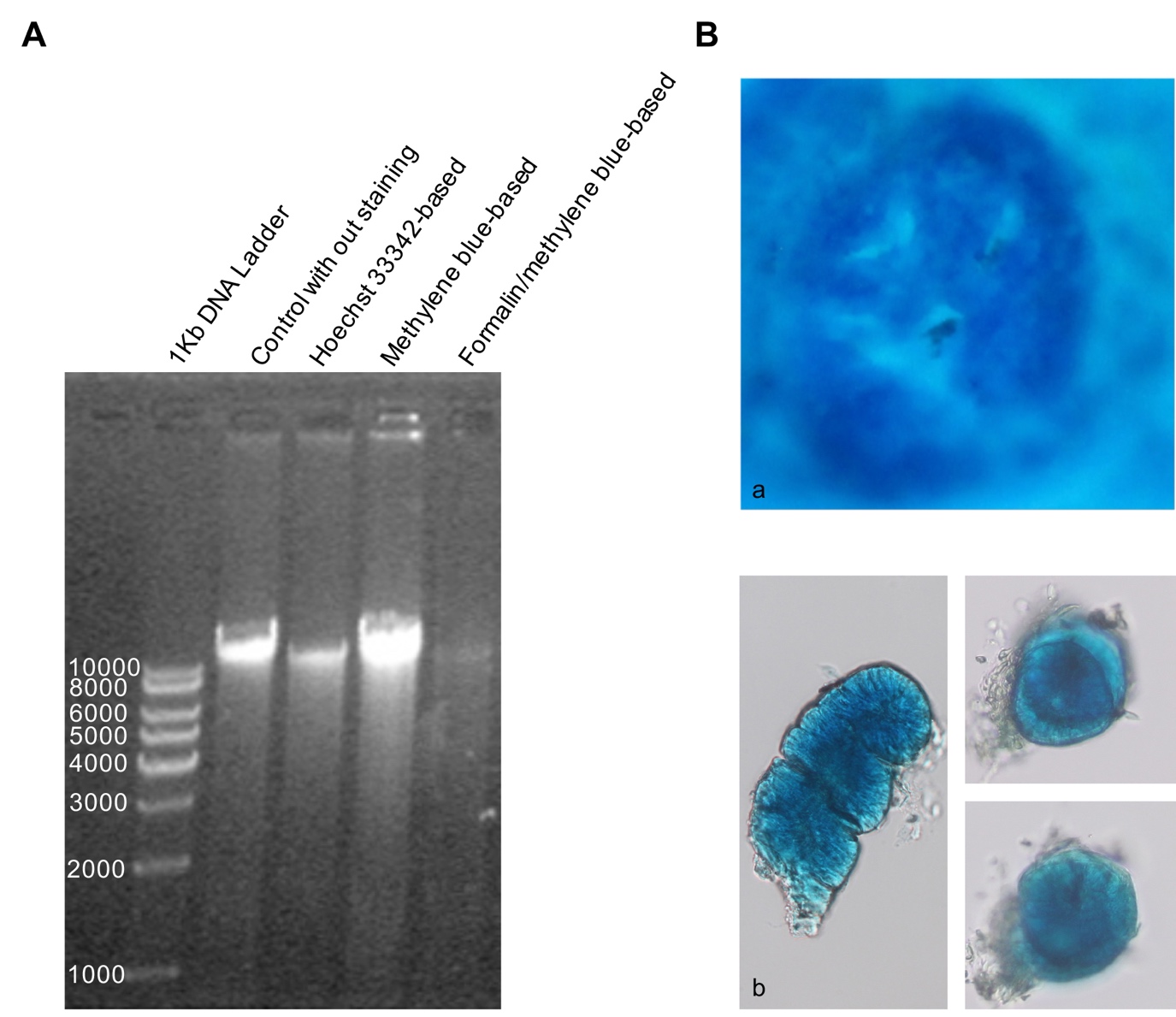


**Supplemental Figure S2. Evaluation of methods for ACF preparation.** (A) Influence of three staining methods (Hoechst 33342-based, methylene blue-based and formalin/methylene blue-based) on DNA quality indicated by agarose gel electrophoresis. Compared with the control, the yield of DNA extraction are much lower by Hoechst 33342 based and formalin/methylene blue-based methods, while for methylene blue-based the yield is relatively consistent. (B) Staining and isolation of ACF. a) ACF identified by methylene blue-based method. b) Three views (side, top and bottom) of single crypt isolated from ACF.


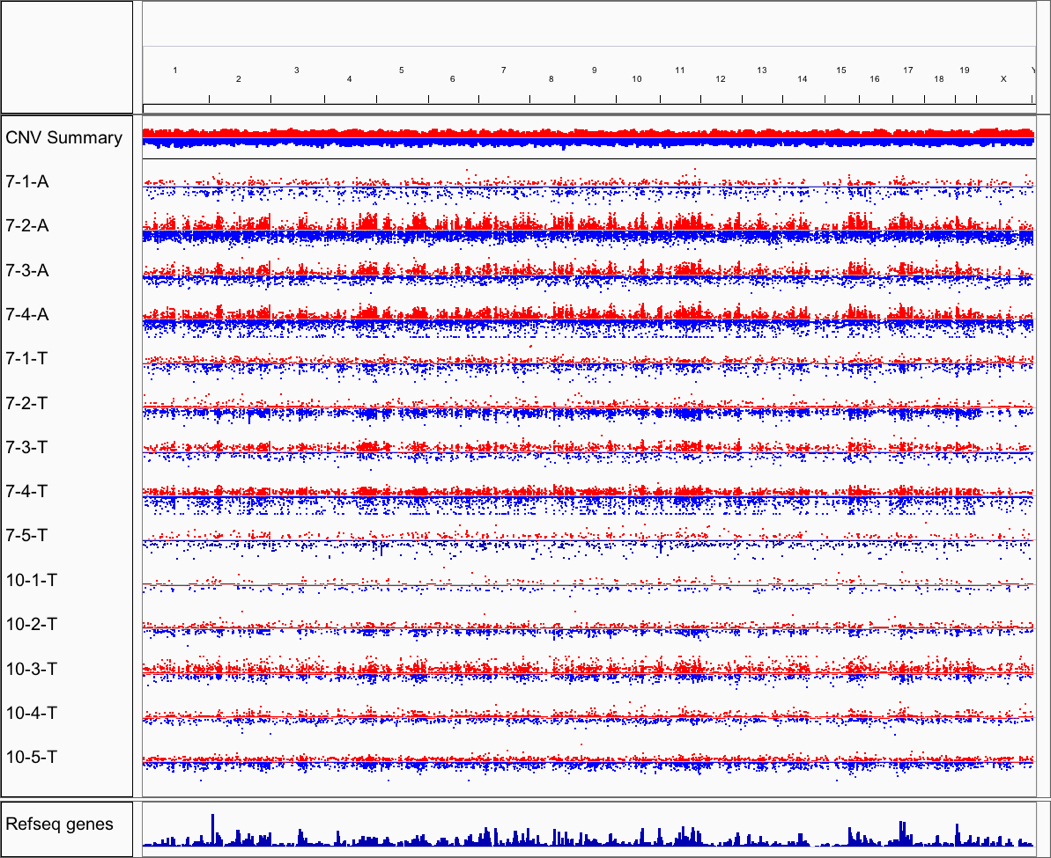


**Supplementary Figure S3 The whole exome profile of CNVs in 14 neoplastic samples of AOM/DSS mouse model.** Log2Ratio of coverage between tumor and paired normal were called by VarScan2, and Circular Binary Segmentation was introduced by DNAcopy R package. The results of all samples were combined by self-built script and visualized by Integrative Genomics Viewer (IGV). Segments with Log2Ratio ranging from -3 to 0 were shown by blue, while those from 0 to 3 were shown by red.


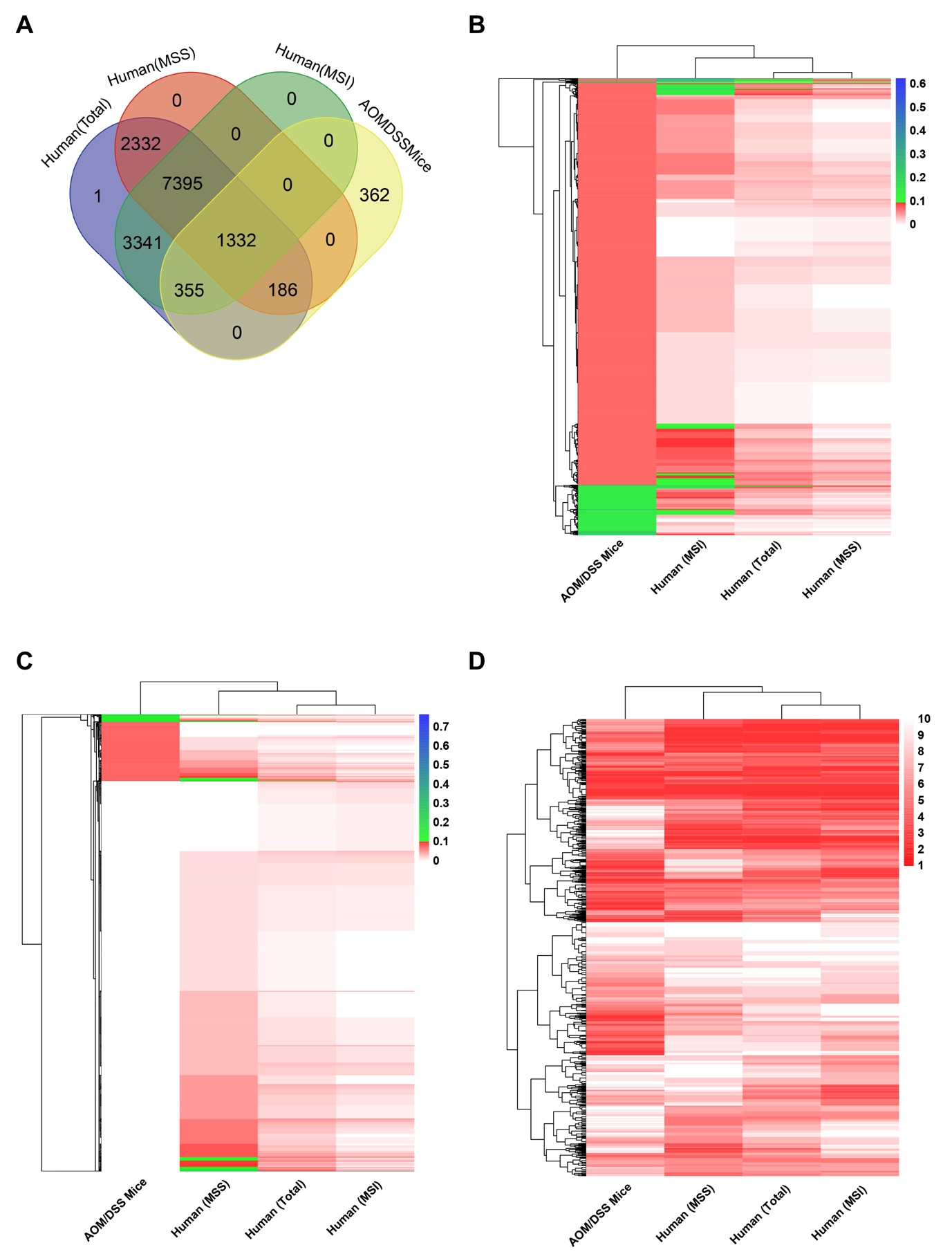


**Supplementary Figure S4** **The profiling of mutation frequencies of somatic mutant genes identified in AOM/DSS mouse model and human CRC.** (A) Venn diagram shows the overlap of somatic mutant genes among mouse model and three human CRC groups. (B) Heat map shows the profiling of mutation frequencies of 15490 somatic mutant genes identified in mouse and human CRC. Since the mutation frequencies of most genes were ranged from 0 to 0.1, a custom legend was made to illustrate the delicate difference of mutation frequencies of these genes properly. Mutation frequencies ranged from 0 to 0.1 are shown with the gradient from white to red, while those over 0.1 are shown with the gradient from green to blue. (C) Heat map shows the profiling of mutation frequencies of 1873 somatic mutated genes identified in both mouse and human CRC. Similar legend was customized. (D) Heat map shows the profiling of ranked mutation frequencies of 1873 somatic mutated genes identified in both mouse and human CRC. Since the sample size of mouse model is much smaller, its pattern is globally hotter than that of three human CRC groups in the heat map generated from the absolute value of mutation frequencies. We corrected this bias by introducing a normalization that all of the 1873 shared genes were divided into 10 ranks according to their frequencies, and heat map was generated from these frequency ranks, instead of the absolute value of frequency.


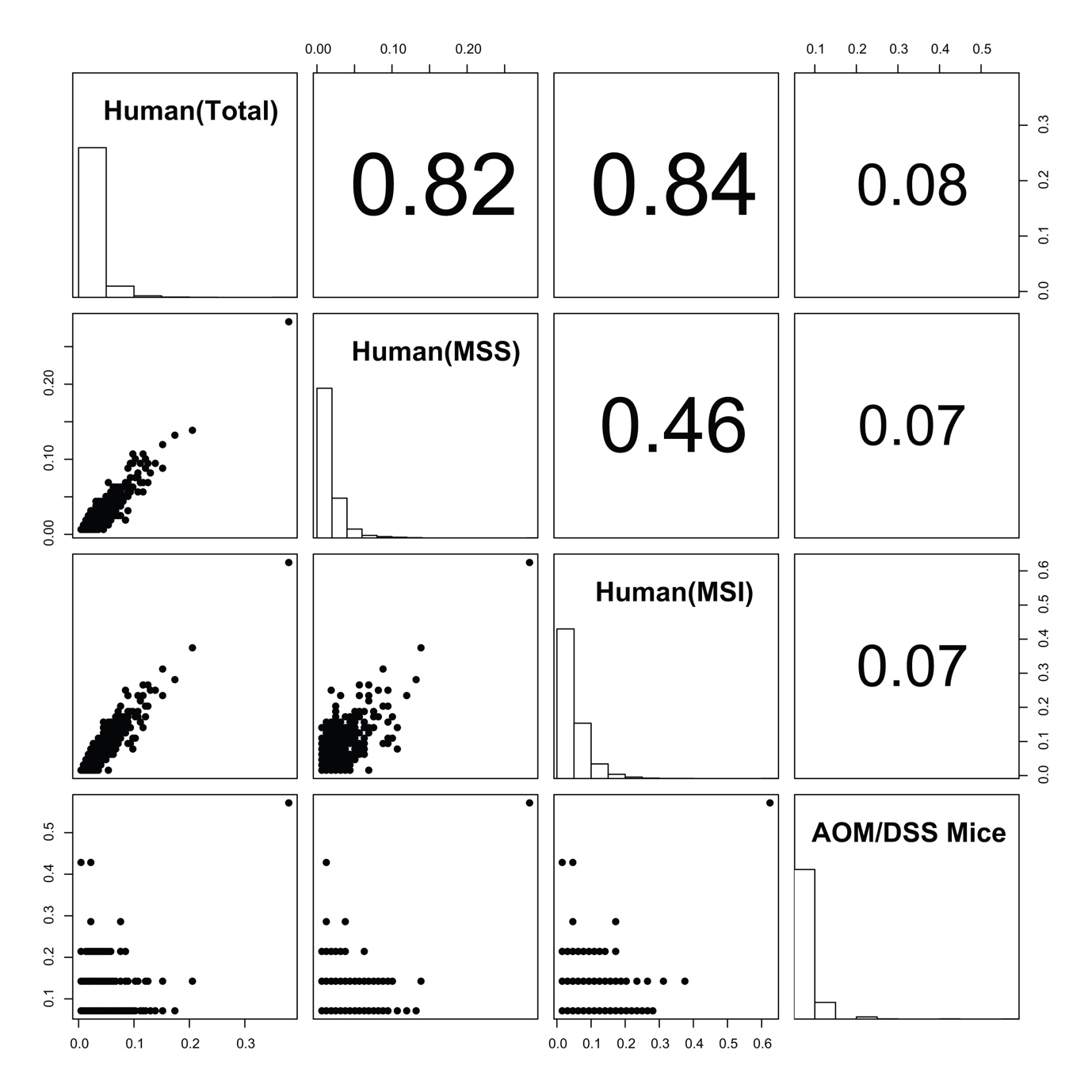


**Supplementary Figure S5** **Scatter plots and Pearson correlations (*R*^2^) of gene frequencies show distinct patterns of somatic mutated genes between AOM/DSS mouse model and three human CRC groups.** In AOM/DSS mouse model, 2235 out of 2509 somatic mutated genes have human orthologs. Among these 2235 genes, there are 1873, 1518 and 1687 genes shared with Total, MSS and MSI of human CRC, respectively. Based on these shared genes, the distribution of mutation frequencies in each group was illustrated by scatter plots, and the similarity of somatic mutated gene patterns between AOM/DSS mouse model and three human CRC groups were quantified by Pearson correlations(*R*^2^). Compared with those defined between human CRC groups (0.82, 0.84 and 0.46), the correlations between mouse model and human CRC (0.08, 0.07 and 0.07) are much lower, indicating that the patterns of somatic mutated genes were quite different in AOM/DSS mouse model and human CRC. In another word, the faithfulness of AOM/DSS mouse model in mimicking human CRC maybe not ideal at the level of gene.


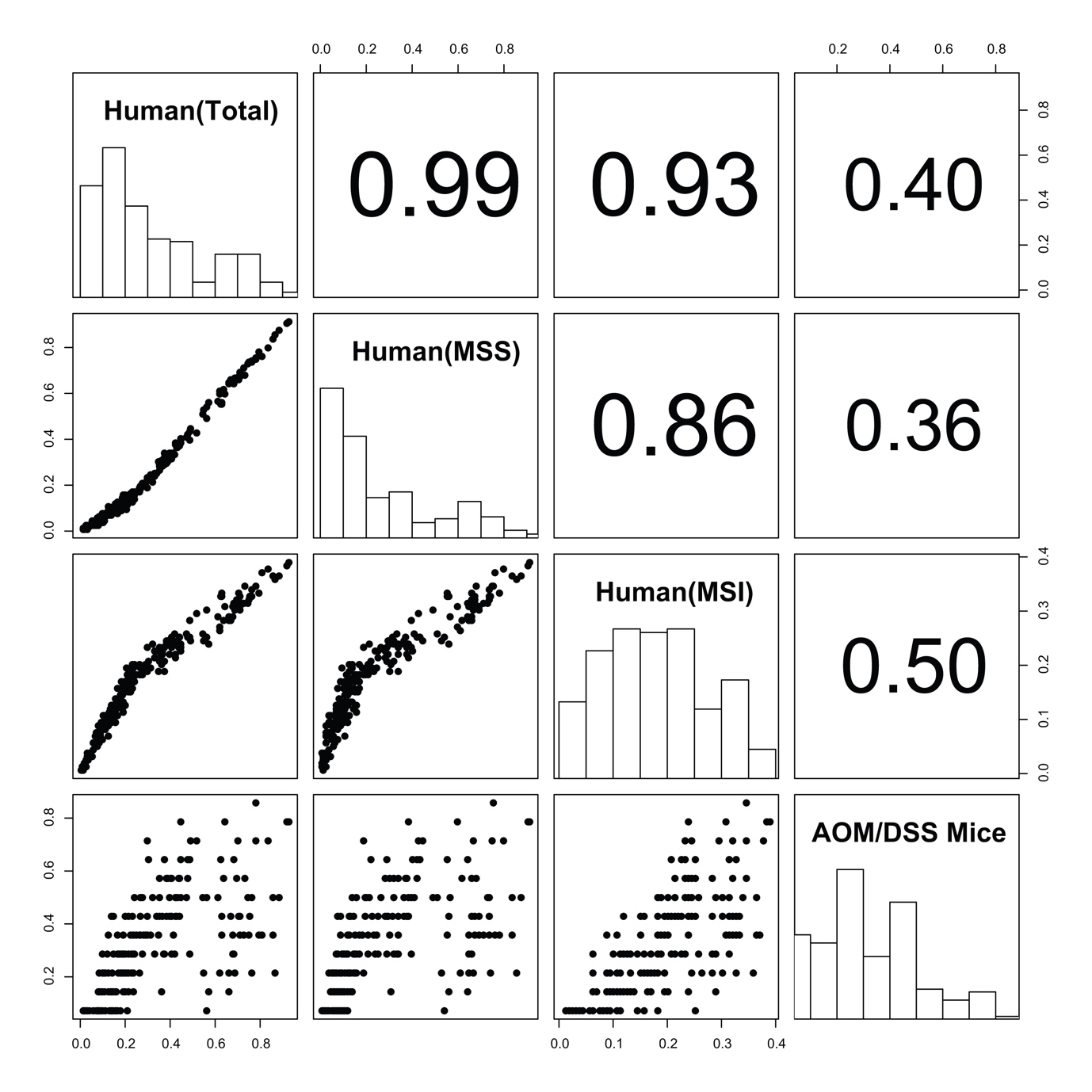


**Supplementary Figure S6** **Scatter plots and Pearson correlations (*R*^2^) of frequencies of perturbed pathways show distinct patterns of pathways perturbation between AOM/DSS mouse model and three human CRC groups.** In AOM/DSS mouse model, 266 KEGG pathways were predicated as perturbed pathways, while 282 KEGG pathways were predicted as perturbed pathways in all of the three human CRC group. Since all of the 266 pathways perturbed in AOM/DSS mouse model were involved in the 282 pathways defined in human CRC, all of the shared 266 pathways were used in next analysis. Based on these shared pathways, the distribution of perturbation frequencies in each group was illustrated by scatter plots, and the similarity of perturbed pathway patterns between AOM/DSS mouse model and three human CRC groups were quantified by Pearson correlations(*R*^2^). Compared with those defined between human CRC groups (0.99, 0.93 and 0.86), the correlations between mouse model and human CRC (0.40, 0.36 and 0.50) are much lower, indicating that the patterns of perturbed pathways were quite different in AOM/DSS mouse model and human CRC. In another word, the faithfulness of AOM/DSS mouse model in mimicking human CRC maybe not ideal at the level of pathway.

**Supplemental Table S1.** The 30 samples prepared for exome sequencing

| **Sample ID** | **Mouse ID** | **Weeks of AOM Induction** | **Cycles of DSS feeding** | | **Tissue Type** | **Tissue Size(mm)** | **Note** |
| --- | --- | --- | --- | --- | --- | --- | --- |
| 7-1-N | 7-1 | 7 | | 2 | Normal | 4 x 3 | - |
| 7-1-A | 7-1 | 7 | | 2 | ACF | - | 6 crypts |
| 7-1-T | 7-1 | 7 | | 2 | Tumor | d=0.8mm | - |
| 7-2-N | 7-2 | 7 | | 2 | Normal | 4.5 x 3 | - |
| 7-2-A | 7-2 | 7 | | 2 | ACF | - | 7 crypts |
| 7-2-T | 7-2 | 7 | | 2 | Tumor | d=0.7mm | - |
| 7-3-N | 7-3 | 7 | | 2 | Normal | 4 x 2.5 | - |
| 7-3-A | 7-3 | 7 | | 2 | ACF | - | 10 crypts |
| 7-3-T | 7-3 | 7 | | 2 | Tumor | d=0.8mm | - |
| 7-4-N | 7-4 | 7 | | 2 | Normal | 4 x 3 | - |
| 7-4-A | 7-4 | 7 | | 2 | ACF | - | 6 crypts |
| 7-4-T | 7-4 | 7 | | 2 | Tumor | d=0.9mm | - |
| 7-5-N | 7-5 | 7 | | 2 | Normal | 4 x 3 | - |
| 7-5-A | 7-5 | 7 | | 2 | ACF | - | 3 crypts |
| 7-5-T | 7-5 | 7 | | 2 | Tumor | d=1mm | - |
| 10-1-N | 10-1 | 10 | | 3 | Normal | 4 x 4 | - |
| 10-1-T | 10-1 | 10 | | 3 | Tumor | d=5.5mm | - |
| 10-2-N | 10-2 | 10 | | 3 | Normal | 4 x 3 | - |
| 10-2-T | 10-2 | 10 | | 3 | Tumor | d=6.3mm | - |
| 10-3-N | 10-3 | 10 | | 3 | Normal | 4 x 3 | - |
| 10-3-T | 10-3 | 10 | | 3 | Tumor | d=7.2mm | - |
| 10-4-N | 10-4 | 10 | | 3 | Normal | 4 x 2.5 | - |
| 10-4-T | 10-4 | 10 | | 3 | Tumor | d=6.3mm | - |
| 10-5-N | 10-5 | 10 | | 3 | Normal | 4 x 3 | - |
| 10-5-T | 10-5 | 10 | | 3 | Tumor | d=7.1mm | - |

**Supplemental Table S2. Coverage statistics across SureSelect target regions and CCDS coordinates of the 30 samples sequenced**

| Sample | | | Reads sequenced | | Reads overlapped with target regions without duplication | | % of reads overlapped with target regions without duplication | | SureSelect target regions | | | | | | CCDS | | | | | |
| --- | --- | --- | --- | --- | --- | --- | --- | --- | --- | --- | --- | --- | --- | --- | --- | --- | --- | --- | --- | --- |
|  |  |  |  |  |  |  |  |  | Depth | | Coverage at 8x | | Coverage at 20x | | Depth | | Coverage at 8X | | Coverage at 20X | |
| 7-1-N | Rep.1 | 96087444 | | 51987248 | | 54.10% | | 207.8 | | 98.98% | | 96.55% | | 194.6 | | 97.48% | | 94.91% | |  |
|  | Rep.2 | 127385736 | | 73387410 | | 57.61% | |  |  |  |  |  |  |  |  |  |  |  |  |  |
| 7-2-N | Rep.1 | 97818762 | | 53672515 | | 54.87% | | 203.5 | | 98.84% | | 96.20% | | 186.6 | | 97.21% | | 94.37% | |  |
|  | Rep.2 | 110741000 | | 52152368 | | 47.09% | |  |  |  |  |  |  |  |  |  |  |  |  |  |
| 7-3-N | Rep.1 | 130602742 | | 65923931 | | 50.48% | | 206.6 | | 98.76% | | 96.09% | | 187.8 | | 97.17% | | 94.30% | |  |
|  | Rep.2 | 92541500 | | 45307478 | | 48.96% | |  |  |  |  |  |  |  |  |  |  |  |  |  |
| 7-4-N | Rep.1 | 106703118 | | 57053561 | | 53.47% | | 158.6 | | 98.02% | | 93.30% | | 145.6 | | 96.33% | | 91.50% | |  |
|  | Rep.2 | 91042740 | | 37118176 | | 40.77% | |  |  |  |  |  |  |  |  |  |  |  |  |  |
| 7-5-N | Rep.1 | 111421292 | | 63781896 | | 57.24% | | 185.9 | | 99.10% | | 96.72% | | 174.1 | | 97.60% | | 95.02% | |  |
|  | Rep.2 | 106586540 | | 46077366 | | 43.23% | |  |  |  |  |  |  |  |  |  |  |  |  |  |
| 7-1-A | Rep.1 | 104530720 | | 62090704 | | 59.40% | | 202.9 | | 98.99% | | 96.64% | | 189.0 | | 97.40% | | 94.86% | |  |
|  | Rep.2 | 84117510 | | 49065235 | | 58.33% | |  |  |  |  |  |  |  |  |  |  |  |  |  |
| 7-2-A | Rep.1 | 117492424 | | 56330871 | | 47.94% | | 231.3 | | 98.87% | | 95.82% | | 217.9 | | 97.33% | | 94.24% | |  |
|  | Rep.2 | 111061508 | | 60413740 | | 54.40% | |  |  |  |  |  |  |  |  |  |  |  |  |  |
| 7-3-A | Rep.1 | 97006348 | | 57384975 | | 59.16% | | 190.0 | | 99.05% | | 96.38% | | 180.6 | | 97.50% | | 94.76% | |  |
|  | Rep.2 | 100419354 | | 53936409 | | 53.71% | |  |  |  |  |  |  |  |  |  |  |  |  |  |
| 7-4-A | Rep.1 | 102024098 | | 52018201 | | 50.99% | | 186.4 | | 98.92% | | 95.96% | | 176.7 | | 97.35% | | 94.33% | |  |
|  | Rep.2 | 93215640 | | 58826923 | | 63.11% | |  |  |  |  |  |  |  |  |  |  |  |  |  |
| 7-5-A | Rep.1 | 91443824 | | 47739425 | | 52.21% | | 149.8 | | 69.63% | | 52.40% | | 146.5 | | 68.30% | | 51.16% | |  |
|  | Rep.2 | 108540086 | | 42789645 | | 39.42% | |  |  |  |  |  |  |  |  |  |  |  |  |  |
| 7-1-T | Rep.1 | 103219530 | | 56181426 | | 54.43% | | 192.4 | | 98.97% | | 96.42% | | 178.2 | | 97.44% | | 94.71% | |  |
|  | Rep.2 | 99647308 | | 49330701 | | 49.51% | |  |  |  |  |  |  |  |  |  |  |  |  |  |
| 7-2-T | Rep.1 | 93624000 | | 52052021 | | 55.60% | | 199.5 | | 98.26% | | 94.59% | | 184.3 | | 96.47% | | 92.61% | |  |
|  | Rep.2 | 96889302 | | 61025023 | | 62.98% | |  |  |  |  |  |  |  |  |  |  |  |  |  |
| 7-3-T | Rep.1 | 106298742 | | 60994675 | | 57.38% | | 200.9 | | 99.19% | | 97.09% | | 188.5 | | 97.65% | | 95.37% | |  |
|  | Rep.2 | 89774470 | | 56180720 | | 62.58% | |  |  |  |  |  |  |  |  |  |  |  |  |  |
| 7-4-T | Rep.1 | 110956256 | | 53494980 | | 48.21% | | 183.7 | | 99.03% | | 96.43% | | 172.9 | | 97.45% | | 94.70% | |  |
|  | Rep.2 | 91903390 | | 55151333 | | 60.01% | |  |  |  |  |  |  |  |  |  |  |  |  |  |
| 7-5-T | Rep.1 | 113817722 | | 66660477 | | 58.57% | | 210.9 | | 99.20% | | 97.21% | | 198.0 | | 97.65% | | 95.44% | |  |
|  | Rep.2 | 90844876 | | 56694396 | | 62.41% | |  |  |  |  |  |  |  |  |  |  |  |  |  |
| 10-1-N | Rep.1 | 98419950 | | 43660493 | | 44.36% | | 206.8 | | 98.89% | | 96.34% | | 191.4 | | 97.31% | | 94.53% | |  |
|  | Rep.2 | 111560408 | | 48754001 | | 43.70% | |  |  |  |  |  |  |  |  |  |  |  |  |  |
| 10-2-N | Rep.1 | 110092386 | | 63470477 | | 57.65% | | 213.3 | | 99.01% | | 96.89% | | 198.4 | | 97.35% | | 95.01% | |  |
|  | Rep.2 | 98645054 | | 58808486 | | 59.62% | |  |  |  |  |  |  |  |  |  |  |  |  |  |
| 10-3-N | Rep.1 | 103705292 | | 56702129 | | 54.68% | | 236.2 | | 99.30% | | 97.71% | | 223.9 | | 97.79% | | 95.90% | |  |
|  | Rep.2 | 130991734 | | 81439588 | | 62.17% | |  |  |  |  |  |  |  |  |  |  |  |  |  |
| 10-4-N | Rep.1 | 99622550 | | 49279435 | | 49.47% | | 257.6 | | 98.04% | | 93.51% | | 235.2 | | 96.37% | | 91.57% | |  |
|  | Rep.2 | 174254226 | | 78722288 | | 45.18% | |  |  |  |  |  |  |  |  |  |  |  |  |  |
| 10-5-N | Rep.1 | 117885188 | | 64975192 | | 55.12% | | 198.1 | | 97.58% | | 92.27% | | 180.7 | | 95.81% | | 90.28% | |  |
|  | Rep.2 | 114616200 | | 47172109 | | 41.16% | |  |  |  |  |  |  |  |  |  |  |  |  |  |
| 10-1-T | Rep.1 | 118840624 | | 50816839 | | 42.76% | | 225.7 | | 99.01% | | 96.67% | | 208.3 | | 97.39% | | 94.83% | |  |
|  | Rep.2 | 106135472 | | 60436642 | | 56.94% | |  |  |  |  |  |  |  |  |  |  |  |  |  |
| 10-2-T | Rep.1 | 99328374 | | 56279131 | | 56.66% | | 197.9 | | 98.77% | | 95.71% | | 182.6 | | 97.15% | | 93.87% | |  |
|  | Rep.2 | 123704046 | | 60126942 | | 48.61% | |  |  |  |  |  |  |  |  |  |  |  |  |  |
| 10-3-T | Rep.1 | 106321178 | | 64049243 | | 60.24% | | 198.7 | | 99.09% | | 96.59% | | 185.4 | | 97.53% | | 94.81% | |  |
|  | Rep.2 | 103565664 | | 62474978 | | 60.32% | |  |  |  |  |  |  |  |  |  |  |  |  |  |
| 10-4-T | Rep.1 | 85781850 | | 52272335 | | 60.94% | | 209.3 | | 97.95% | | 92.97% | | 192.4 | | 96.22% | | 91.05% | |  |
|  | Rep.2 | 122894624 | | 72802618 | | 59.24% | |  |  |  |  |  |  |  |  |  |  |  |  |  |
| 10-5-T | Rep.1 | 197384512 | | 80812159 | | 40.94% | | 312.9 | | 98.90% | | 96.45% | | 285.4 | | 97.39% | | 94.58% | |  |
|  | Rep.2 | 157387948 | | 77162764 | | 49.03% | |  |  |  |  |  |  |  |  |  |  |  |  |  |

**Supplemental Table S3.** The somatic mutations identified in 14 neoplastic samples of AOM/DSS mouse model. (This table is too large, and hence submitted separately.)

**Supplemental Table S4.** The profiling of mutant genes in AOM/DSS mouse model. (This table is too large, and hence submitted separately.)

**Supplemental Table S5.** The profiling of perturbed KEGG pathways in AOM/DSS mouse model. (This table is too large, and hence submitted separately.)

**Supplemental Table S6.** mm9-based gene mutation table for identifying significantly mutated genes by DrGap. (This table is too large, and hence submitted separately.)

**Supplemental Table S7.** SRS and BioSample IDs for all samples analyzed

| **Sample ID** | **SRS**  **ID** | **BioSample**  **ID** | **Phenotype** | **Accession of technical replicates** | |
| --- | --- | --- | --- | --- | --- |
|  |  |  |  | **Replicate 1** | **Replicate 2** |
| 0-1-C | SRS1174603 | SAMN04286860 | Normal mucosa | SRX1452751 | SRX1452752 |
| 0-2-C | SRS1179216 | SAMN04286862 | Normal mucosa | SRX1462096 | SRX1462097 |
| 0-3-C | SRS1180553 | SAMN04286864 | Normal mucosa | SRX1452858 | SRX1452859 |
| 0-4-C | SRS1179608 | SAMN04286866 | Normal mucosa | SRX1451723 | SRX1451767 |
| 0-5-C | SRS1180392 | SAMN04286868 | Normal mucosa | SRX1452664 | SRX1452665 |
| 7-1-N | SRS1180393 | SAMN04286870 | Normal mucosa | SRX1452666 | SRX1452667 |
| 7-2-N | SRS1180426 | SAMN04286876 | Normal mucosa | SRX1452700 | SRX1452701 |
| 7-3-N | SRS1180427 | SAMN04286882 | Normal mucosa | SRX1452702 | SRX1452703 |
| 7-4-N | SRS1180428 | SAMN04286888 | Normal mucosa | SRX1452704 | SRX1452705 |
| 7-5-N | SRS1180429 | SAMN04286894 | Normal mucosa | SRX1452706 | SRX1452707 |
| 7-1-A | SRS1180430 | SAMN04286872 | Aberrant crypt foci | SRX1452708 | SRX1452709 |
| 7-2-A | SRS1180431 | SAMN04286878 | Aberrant crypt foci | SRX1452710 | SRX1452712 |
| 7-3-A | SRS1180433 | SAMN04286884 | Aberrant crypt foci | SRX1452713 | SRX1452714 |
| 7-4-A | SRS1180434 | SAMN04286890 | Aberrant crypt foci | SRX1452715 | SRX1452716 |
| 7-5-A | SRS1180435 | SAMN04286896 | Aberrant crypt foci | SRX1452717 | SRX1452718 |
| 7-1-T | SRS1180436 | SAMN04286874 | Tumor(d≤1mm) | SRX1452719 | SRX1452720 |
| 7-2-T | SRS1180437 | SAMN04286880 | Tumor(d≤1mm) | SRX1452721 | SRX1452722 |
| 7-3-T | SRS1180438 | SAMN04286886 | Tumor(d≤1mm) | SRX1452723 | SRX1452724 |
| 7-4-T | SRS1180439 | SAMN04286892 | Tumor(d≤1mm) | SRX1452725 | SRX1452726 |
| 7-5-T | SRS1180440 | SAMN04286898 | Tumor(d≤1mm) | SRX1452727 | SRX1452728 |
| 10-1-N | SRS1180441 | SAMN04286900 | Normal mucosa | SRX1452729 | SRX1452730 |
| 10-2-N | SRS1180444 | SAMN04286904 | Normal mucosa | SRX1452733 | SRX1452734 |
| 10-3-N | SRS1180445 | SAMN04286908 | Normal mucosa | SRX1452735 | SRX1452736 |
| 10-4-N | SRS1180446 | SAMN04286912 | Normal mucosa | SRX1452737 | SRX1452738 |
| 10-5-N | SRS1180447 | SAMN04286916 | Normal mucosa | SRX1452739 | SRX1452740 |
| 10-1-T | SRS1180448 | SAMN04286902 | Tumor(d>5mm) | SRX1452741 | SRX1452742 |
| 10-2-T | SRS1180449 | SAMN04286906 | Tumor(d>5mm) | SRX1452743 | SRX1452744 |
| 10-3-T | SRS1180450 | SAMN04286910 | Tumor(d>5mm) | SRX1452745 | SRX1452746 |
| 10-4-T | SRS1180451 | SAMN04286914 | Tumor(d>5mm) | SRX1452747 | SRX1452748 |
| 10-5-T | SRS1180452 | SAMN04286918 | Tumor(d>5mm) | SRX1452749 | SRX1452750 |
